# Supplementary material for: The Small RNA Universe of Capitella teleta
Source: Front Mol Biosci. 2022 Feb 25;9:802814. doi: 10.3389/fmolb.2022.802814 (PMC8915122; doi:10.3389/fmolb.2022.802814)
Supplement: Supplementary file 1 [file DataSheet1.ZIP › Supplement/known/CAPTEscaffold_300_13329.pdf]

Provisional ID : CAPTEscaffold_300_13329
Score total : 65761.5
Score for star read(s) : 3.9
Score for read counts : 65755.8
Score for mfe : 0.8
Score for randfold : 1.6
Score for cons. seed : -0.6
Total read count : 128989
Mature read count : 97026
Loop read count : 0
Star read count : 31963

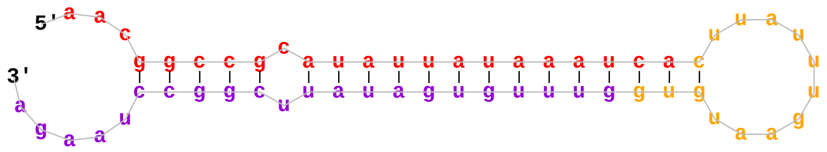

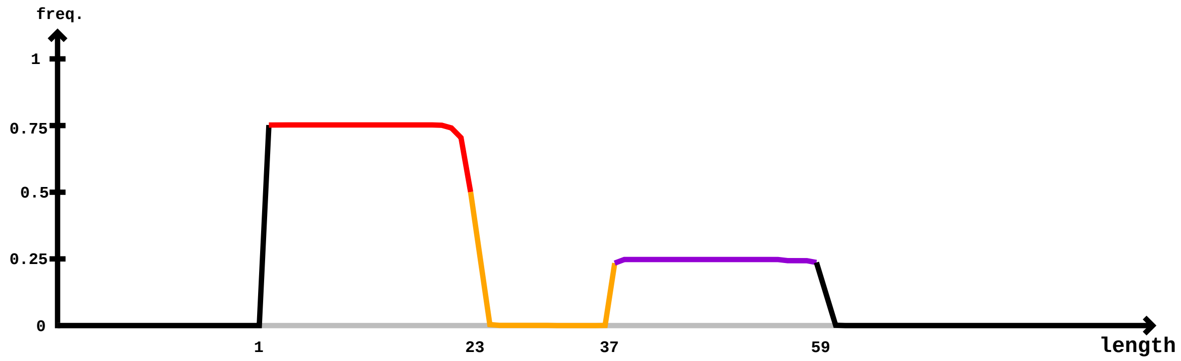

Mature **Star**

```
5'- auguuuucucgcgugaaaucaacggccgcauauuauaaaucacuuauuugaauguggguuugugauauucggccuaagauuaccacucguaaugcugcuaacaauaucugauu   -3'   obs
    auguuuucucgcgugaaaucaacggccgcauauuauaaaucacuuauuugaauguggguuugugauauucggccuaagauuaccacucguaaugcugcuaacaauaucugauu         exp
    auguuuucucgcgugaaaucaacggccgcauauuauaaaucacuuauuugaauguggguuugugauauucggccuaagauuaccacucguaaugcugcuaacaauaucugauu         known
    ..((.....((.(((.((((...(((((.((((((((((((((.........)))))))))))))).)))))...))))..))).))....))...................   reads   mm   sample

    ...................caacggccgcauauuauaaauc.......................................................................   4   0   seq
    ...................caacggccgcauauuauaaauca......................................................................   1   0   seq
    ....................aacggAcgcauauuauaa..........................................................................   1   1   seq
    ....................aacggccgcauauuauaa..........................................................................   162   0   seq
    ....................Gacggccgcauauuauaaa.........................................................................   2   1   seq
    ....................aGcggccgcauauuauaaa.........................................................................   1   1   seq
    ....................aacUgccgcauauuauaaa.........................................................................   2   1   seq
    ....................aacggccgcauauuauaaa.........................................................................   1284   0   seq
    ....................aacggccUcauauuauaaa.........................................................................   1   1   seq
    ....................aacggcUgcauauuauaaa.........................................................................   2   1   seq
    ....................aacggccgcauauuauaUa.........................................................................   1   1   seq
    ....................aacggccgcUuauuauaaa.........................................................................   1   1   seq
    ....................aacggccgcauauuauaaG.........................................................................   1   1   seq
    ....................aacggccgcaAauuauaaa.........................................................................   1   1   seq
    ....................aacggccAcauauuauaaa.........................................................................   1   1   seq
    ....................aacggAcgcauauuauaaa.........................................................................   2   1   seq
    ....................aacAgccgcauauuauaaa.........................................................................   1   1   seq
    ....................aacggccgcauauuauaaU.........................................................................   1   1   seq
    ....................Nacggccgcauauuauaaa.........................................................................   1   1   seq
    ....................aacggccgcauauuauaaaC........................................................................   1   1   seq
    ....................aacggcUgcauauuauaaau........................................................................   4   1   seq
    ....................aacggccgcauauuauaaaA........................................................................   11   1   seq
    ....................aacggccgGauauuauaaau........................................................................   1   1   seq
    ....................aacggAcgcauauuauaaau........................................................................   3   1   seq
    ....................aacggccgcauauuauaGau........................................................................   1   1   seq
    ....................aacUgccgcauauuauaaau........................................................................   1   1   seq
    ....................aacggccgcaAauuauaaau........................................................................   2   1   seq
    ....................aacggccgcauauuaCaaau........................................................................   1   1   seq
    ....................aacggccgcauauAauaaau........................................................................   2   1   seq
    ....................aacggccgcauauuauaUau........................................................................   1   1   seq
    ....................aacAgccgcauauuauaaau........................................................................   3   1   seq
```

```
                    Mature                            Star
auguuuucucgcgugaaaucaacggccgcauauuauaaaucacuuauuuugaauguguuugugauauucggccuaagauuaccacucguaaugcugcuaacaauaucugauu
....................aaAggccgcauauuauaaau.........................................................................   1      1    seq
....................aaUggccgcauauuauaaau.........................................................................   1      1    seq
....................aacggccgcauaAuauaaau.........................................................................   1      1    seq
....................aGcggccgcauauuauaaau.........................................................................   1      1    seq
....................aUcggccgcauauuauaaau.........................................................................   1      1    seq
....................aacggccgcauauuauUaau.........................................................................   2      1    seq
....................Gacggccgcauauuauaaau.........................................................................   5      1    seq
....................aacggccgcauauuauaaau.........................................................................   4635   0    seq
....................aacgUccgcauauuauaaau.........................................................................   1      1    seq
....................aacggccCcauauuauaaau.........................................................................   2      1    seq
....................aacggccAcauauuauaaau.........................................................................   2      1    seq
....................aacggcGgcauauuauaaau.........................................................................   1      1    seq
....................aacggccgAauauuauaaau.........................................................................   2      1    seq
....................Nacggccgcauauuauaaau.........................................................................   2      1    seq
....................aacggccgcauauuUuaaau.........................................................................   1      1    seq
....................Cacggccgcauauuauaaau.........................................................................   1      1    seq
....................aacggcAgcauauuauaaau.........................................................................   7      1    seq
....................aacggccgcauGuuauaaau.........................................................................   1      1    seq
....................aacggccgcauauuGuaaau.........................................................................   2      1    seq
....................aacggccgcauauuauaaaG.........................................................................   2      1    seq
....................aacggccgcaCauuauaaauc........................................................................   5      1    seq
....................aacggAcgcauauuauaaauc........................................................................   22     1    seq
....................aacggccgcauauuauaaaCc........................................................................   2      1    seq
....................aacgAccgcauauuauaaauc........................................................................   4      1    seq
....................aacggcUgcauauuauaaauc........................................................................   4      1    seq
....................aacCgccgcauauuauaaauc........................................................................   1      1    seq
....................aacggcGgcauauuauaaauc........................................................................   3      1    seq
....................aaUggccgcauauuauaaauc........................................................................   2      1    seq
....................aGcggccgcauauuauaaauc........................................................................   2      1    seq
....................aacAgccgcauauuauaaauc........................................................................   11     1    seq
....................aacggccgcCuauuauaaauc........................................................................   1      1    seq
....................aUcggccgcauauuauaaauc........................................................................   1      1    seq
....................Uacggccgcauauuauaaauc........................................................................   2      1    seq
....................aacggccgcauauuauaaauA........................................................................   210    1    seq
....................aacggccUcauauuauaaauc........................................................................   6      1    seq
....................aacUgccgcauauuauaaauc........................................................................   2      1    seq
....................aacggcAgcauauuauaaauc........................................................................   46     1    seq
....................Cacggccgcauauuauaaauc........................................................................   7      1    seq
....................aacggccgcauauAauaaauc........................................................................   3      1    seq
....................aacggccgcauaAuauaaauc........................................................................   8      1    seq
....................aacgCccgcauauuauaaauc........................................................................   2      1    seq
....................aacggccgcGuauuauaaauc........................................................................   3      1    seq
....................aacggccgcauauCauaaauc........................................................................   2      1    seq
....................aacggccgcauauuauaGauc........................................................................   4      1    seq
....................aacggGcgcauauuauaaauc........................................................................   2      1    seq
....................Gacggccgcauauuauaaauc........................................................................   16     1    seq
....................aacggccgcauauuauUaauc........................................................................   1      1    seq
....................aacggccgcauauuGuaaauc........................................................................   3      1    seq
....................aacggccgcauauuauaaaAc........................................................................   7      1    seq
....................aacggccgAauauuauaaauc........................................................................   7      1    seq
....................aacggccgcauauuauGaauc........................................................................   2      1    seq
....................aacggccgcauauuAaaauc.........................................................................   17     1    seq
....................aacggccgcauauuauaaGuc........................................................................   3      1    seq
....................aacggccgUauauuauaaauc........................................................................   448    1    seq
....................aacggNcgcauauuauaaauc........................................................................   1      1    seq
....................aacggccgcauauuauaaaGc........................................................................   1      1    seq
....................aacggccgcUuauuauaaauc........................................................................   4      1    seq
....................aaGggccgcauauuauaaauc........................................................................   2      1    seq
....................aacggccgcauauuauaaauc........................................................................   25369  0    seq
....................aacggccgcauauuUuaaauc........................................................................   3      1    seq
....................aacggccgcauauuaGaaauc........................................................................   1      1    seq
....................aacggccgcauUuuauaaauc........................................................................   2      1    seq
....................aacggUcgcauauuauaaauc........................................................................   3      1    seq
....................aacggccgcauauuaCaaauc........................................................................   5      1    seq
....................aacggccgcaGauuauaaauc........................................................................   3      1    seq
....................aacggccgcauaGuauaaauc........................................................................   1      1    seq
....................aacggccgcauauuauaaUuc........................................................................   2      1    seq
....................Nacggccgcauauuauaaauc........................................................................   7      1    seq
....................aacggccgcauauuauaUauc........................................................................   2      1    seq
....................aacggccgcauauuauaaauG........................................................................   3      1    seq
```

```
                    Mature                              Star
auguuuucucgcgugaaaucaacggccgcauauuauaaaucacuuauuugaauguguuugugauauucggccuaagauuaccacucguaaugcugcuaacaauaucugauu
....................aaAggccgcauauuauaaauc.......................................................................    8      1    seq
....................aacggccCcauauuauaaauc.......................................................................    5      1    seq
....................aacggccAcauauuauaaauc.......................................................................    11     1    seq
....................aacggccgcaAauuauaaauc.......................................................................    6      1    seq
....................aCcggccgcauauuauaaauc.......................................................................    1      1    seq
....................aacggccgcauGuuauaaauca......................................................................    5      1    seq
....................aUcggccgcauauuauaaauca......................................................................    3      1    seq
....................aacggccgcauauuaCaaauca......................................................................    6      1    seq
....................aacggccgcauUuuauaaauca......................................................................    4      1    seq
....................aacggccgcauauuauaaaAca......................................................................    7      1    seq
....................aacUgccgcauauuauaaauca......................................................................    1      1    seq
....................aacggccgcauauuauUaauca......................................................................    3      1    seq
....................Uacggccgcauauuauaaauca......................................................................    3      1    seq
....................aacggccgAauauuauaaauca......................................................................    22     1    seq
....................aacggccgcauauCauaaauca......................................................................    5      1    seq
....................aacggGcgcauauuauaaauca......................................................................    1      1    seq
....................aacCgccgcauauuauaaauca......................................................................    5      1    seq
....................aNcggccgcauauuauaaauca......................................................................    2      1    seq
....................aacggccgUauauuauaaauca......................................................................    46     1    seq
....................aacggccgcauauuauaUauca......................................................................    2      1    seq
....................aacggccgcauauuauaaauGa......................................................................    1      1    seq
....................aaUggccgcauauuauaaauca......................................................................    8      1    seq
....................aacggccgcUuauuauaaauca......................................................................    8      1    seq
....................aacggccgcauauuauaaaucU......................................................................    112    1    seq
....................aacggccgcaAauuauaaauca......................................................................    27     1    seq
....................aacggccgcauauuaAaaauca......................................................................    24     1    seq
....................Gacggccgcauauuauaaauca......................................................................    38     1    seq
....................aacgAccgcauauuauaaauca......................................................................    11     1    seq
....................aacggNcgcauauuauaaauca......................................................................    1      1    seq
....................aacggccCcauauuauaaauca......................................................................    4      1    seq
....................aacggccgcauauuauaaauca......................................................................    53974  0    seq
....................aGcggccgcauauuauaaauca......................................................................    7      1    seq
....................aacggUcgcauauuauaaauca......................................................................    6      1    seq
....................aacggccUcauauuauaaauca......................................................................    10     1    seq
....................Nacggccgcauauuauaaauca......................................................................    13     1    seq
....................aacggccgGauauuauaaauca......................................................................    5      1    seq
....................aacggccgcauauuauaaauAa......................................................................    17     1    seq
....................aacggccgcauauuauaGauca......................................................................    13     1    seq
....................aacggccAcauauuauaaauca......................................................................    11     1    seq
....................Cacggccgcauauuauaaauca......................................................................    17     1    seq
....................aacggccgcauauuauaaauUa......................................................................    24     1    seq
....................aacggccgcauaCuauaaauca......................................................................    2      1    seq
....................aacggccgcauauuauaaGuca......................................................................    8      1    seq
....................aacggcUgcauauuauaaauca......................................................................    16     1    seq
....................aacggccgcauauuauaaCuca......................................................................    1      1    seq
....................aCcggccgcauauuauaaauca......................................................................    4      1    seq
....................aaGggccgcauauuauaaauca......................................................................    1      1    seq
....................aacggccgcauauuauGaauca......................................................................    9      1    seq
....................aacggcGgcauauuauaaauca......................................................................    1      1    seq
....................aacgUccgcauauuauaaauca......................................................................    7      1    seq
....................aacAgccgcauauuauaaauca......................................................................    23     1    seq
....................aacggccgcauauAauaaauca......................................................................    10     1    seq
....................aacggccgcauauuauaaUuca......................................................................    4      1    seq
....................aacggccgcauauuGuaaauca......................................................................    9      1    seq
....................aaAggccgcauauuauaaauca......................................................................    18     1    seq
....................aacggccgcaCauuauaaauca......................................................................    3      1    seq
....................aacggcAgcauauuauaaauca......................................................................    108    1    seq
....................aacggccgcauauuauCaauca......................................................................    1      1    seq
....................aacgCccgcauauuauaaauca......................................................................    2      1    seq
....................aacggccgNauauuauaaauca......................................................................    1      1    seq
....................aacggccgcaGauuauaaauca......................................................................    2      1    seq
....................aacggccgcauauuauaaaucG......................................................................    114    1    seq
....................aacggAcgcauauuauaaauca......................................................................    61     1    seq
....................aacggccgcauauuauaaaucC......................................................................    3      1    seq
....................aacggccgcGuauuauaaauca......................................................................    5      1    seq
....................aacggccgcauauuUuaaauca......................................................................    8      1    seq
....................aacggccgcauauuauaaaCca......................................................................    3      1    seq
....................aacggccgcauaAuauaaauca......................................................................    16     1    seq
....................aacggccgcauauuauaaaGca......................................................................    6      1    seq
....................aGcggccgcauauuauaaaucac.....................................................................    2      1    seq
```

Mature Star			
auguuuucucgcgugaaaucaacggccgcauauuauaaaucacuuauuugaaugugguuugugauauucggccuaagauuaccacucguaaugcugcuaacaauaucugauu			
....................aacggccgcaAauuauaaaucac...	5	1	seq
....................aacggccgcGuauuauaaaucac...	2	1	seq
....................aacggccgcauauuauaaaucCc...	1	1	seq
....................aacggccgcauauuauaaaucac...	8877	0	seq
....................aacggcAgcauauuauaaaucac...	18	1	seq
....................aacggccAcauauuauaaaucac...	1	1	seq
....................aacggcUgcauauuauaaaucac...	3	1	seq
....................Nacggccgcauauuauaaaucac...	1	1	seq
....................aacggccgcauauuauaaaAcac...	1	1	seq
....................aacggccgcauauuauaaaucaU...	11	1	seq
....................aacggccCcauauuauaaaucac...	1	1	seq
....................aacggNcgcauauuauaaaucac...	1	1	seq
....................aacgUccgcauauuauaaaucac...	1	1	seq
....................aacggccgGauauuauaaaucac...	1	1	seq
....................aacggccgAauauuauaaaucac...	4	1	seq
....................aUcggccgcauauuauaaaucac...	1	1	seq
....................aacggAcgcauauuauaaaucac...	7	1	seq
....................aacggccgcauauuauaGaucac...	3	1	seq
....................aacggccgcauauuauaaaucUc...	3	1	seq
....................aaAggccgcauauuauaaaucac...	5	1	seq
....................aacggccgcauauuauaaUucac...	1	1	seq
....................aacggccgcauauuaAaaaucac...	4	1	seq
....................aacggccgcauauuauGaaucac...	4	1	seq
....................aacggUcgcauauuauaaaucac...	1	1	seq
....................aacggccgcauaCuauaaaucac...	1	1	seq
....................Cacggccgcauauuauaaaucac...	4	1	seq
....................aacggccgcauauuauaaaucaG...	1	1	seq
....................aacggccgcauauuaCaaaucac...	1	1	seq
....................aCcggccgcauauuauaaaucac...	1	1	seq
....................aacggccgcauauuauaaauAac...	4	1	seq
....................aacggccgcauauuUuaaaucac...	1	1	seq
....................Gacggccgcauauuauaaaucac...	6	1	seq
....................aacgAccgcauauuauaaaucac...	2	1	seq
....................aacAgccgcauauuauaaaucac...	1	1	seq
....................aacggccgcauauuauUaaucac...	3	1	seq
....................aacggccgcauauuauaaauUac...	3	1	seq
....................aaUggccgcauauuauaaaucac...	2	1	seq
....................aacggcGgcauauuauaaaucac...	2	1	seq
....................aacggccgcauaAuauaaaucac...	2	1	seq
....................aacggccgcauauuauaaaucaA...	194	1	seq
....................aacggccgcauauuauaaaucacC..	2	1	seq
....................aacggccgcauauuauaaaucacA..	6	1	seq
....................aacggccgcauauuauaaaucaUu..	47	1	seq
....................aacCgccgcauauuauaaaucacu..	1	1	seq
....................aacggccgcauauuauaaaucacu..	371	0	seq
....................aacggccgcauauuauaaaucacuu...	1	0	seq
....................aacggccgcauauuauaaaucacuuauuug..	78	0	seq
.....................acggccgcauauuauaaau..	1	0	seq
.....................acCgccgcauauuauaaau..	1	1	seq
.....................acggccgcauauuauaaauc...	8	0	seq
.....................acggccgcauauuauaaauA...	3	1	seq
.....................aAggccgcauauuauaaauca..	1	1	seq
.....................acggccgcauauuauaaauAa..	4	1	seq
.....................acggccgcaAauuauaaauca..	1	1	seq
.....................acggccgcauauuauaaauca..	2	0	seq
......................cggccgcauauuauaaauca..	27	0	seq
....augugguuugugauauucggccua.....................................	5	0	seq
....ugugguuugugauauucggccu......................................	1	0	seq
....gguuugugauauucggcc.......................................	5	0	seq
....gguuugugauauucggccu......................................	2	0	seq
....gguuugugauauucggccuaaA...................................	1	1	seq
....gguuugugauauucggccuaag...................................	40	0	seq
....gguuugugauauucggccuaaga..................................	1	0	seq
....guuugugauGuucggccu......................................	1	1	seq
....guuugugauauucggccA......................................	1	1	seq
....guuugugauauucggccu......................................	542	0	seq
....guuAgugauauucggccu......................................	1	1	seq
....guuugugauauucggccG......................................	2	1	seq
....gAuugugauauucggccu......................................	1	1	seq
....Uuuugugauauucggccu......................................	1	1	seq

```
                    Mature                              Star
auguuuucucgcgugaaaucaacggccgcauauuauaaaucacuuauuugaauguguuugugauauucggccuaagauuaccacucguaaugcuacaauaucugauu
........................................................guuugugauauucggccua.................................  4      0  seq
........................................................guuugugauauucggccuaa................................  4      0  seq
........................................................gAuugugauauucggccuaag...............................  1      1  seq
........................................................guuugugauauucggccuGag...............................  1      1  seq
........................................................guuugugGuauucggccuaag...............................  1      1  seq
........................................................gGuugugauauucggccuaag...............................  2      1  seq
........................................................guuugCgauauucggccuaag...............................  1      1  seq
........................................................guuugugauauuAggccuaag...............................  1      1  seq
........................................................guuugugauauucggccuaGg...............................  1      1  seq
........................................................guuugugauauucggccuaag...............................  790    0  seq
........................................................guuugugauauuUggccuaag...............................  2      1  seq
........................................................guuugugaAauucggccuaag...............................  1      1  seq
........................................................Uuuugugauauucggccuaag...............................  2      1  seq
........................................................guAugugauauucggccuaag...............................  1      1  seq
........................................................gAuugugauauucggccuaaga..............................  15     1  seq
........................................................guuugugauauucggccuaagG..............................  12     1  seq
........................................................guuugugauauucggcUuaaga..............................  9      1  seq
........................................................guuuCugauauucggccuaaga..............................  1      1  seq
........................................................gGuugugauauucggccuaaga..............................  26     1  seq
........................................................guuuguAauauucggccuaaga..............................  7      1  seq
........................................................guuugugauauuGggccuaaga..............................  4      1  seq
........................................................guuugugauauucggAcuaaga..............................  3      1  seq
........................................................guuugugauauAcggccuaaga..............................  12     1  seq
........................................................guuugugauauucggGcuaaga..............................  2      1  seq
........................................................guuCgugauauucggccuaaga..............................  4      1  seq
........................................................guuugugauauucggccuaagU..............................  105    1  seq
........................................................guuugugauauucggccuaGga..............................  3      1  seq
........................................................guuugugauauucggcGuaaga..............................  1      1  seq
........................................................guuugAgauauucggccuaaga..............................  12     1  seq
........................................................Cuuugugauauucggccuaaga..............................  5      1  seq
........................................................guuugugauaCucggccuaaga..............................  1      1  seq
........................................................guCugugauauucggccuaaga..............................  2      1  seq
........................................................guuugugauauucggccuaaCa..............................  1      1  seq
........................................................guuuUugauauucggccuaaga..............................  2      1  seq
........................................................guuugugauauCcggccuaaga..............................  1      1  seq
........................................................guuugugauGuucggccuaaga..............................  13     1  seq
........................................................guuugugauauucggccuaaga..............................  27762  0  seq
........................................................guAugugauauucggccuaaga..............................  20     1  seq
........................................................guuugCgauauucggccuaaga..............................  2      1  seq
........................................................guuAgugauauucggccuaaga..............................  9      1  seq
........................................................guuugugauauucAgccuaaga..............................  8      1  seq
........................................................Uuuugugauauucggccuaaga..............................  35     1  seq
........................................................guuugugauUuucggccuaaga..............................  3      1  seq
........................................................guuugugauauucggccuaaAa..............................  4      1  seq
........................................................guuuguUauauucggccuaaga..............................  4      1  seq
........................................................guuuAugauauucggccuaaga..............................  9      1  seq
........................................................Auuugugauauucggccuaaga..............................  4      1  seq
........................................................Nuuugugauauucggccuaaga..............................  9      1  seq
........................................................guuugugauauucggccCaaga..............................  2      1  seq
........................................................guuugugGuauucggccuaaga..............................  2      1  seq
........................................................guuugugauauuUggccuaaga..............................  57     1  seq
........................................................guuugugauauucggcAuaaga..............................  2      1  seq
........................................................guuugugauaAucggccuaaga..............................  7      1  seq
........................................................guuugugaGauucggccuaaga..............................  2      1  seq
........................................................guuugugauauuAggccuaaga..............................  10     1  seq
........................................................guuugugaCauucggccuaaga..............................  4      1  seq
........................................................guuugugauauucggccAaaga..............................  1      1  seq
........................................................guuugugauauucggUcuaaga..............................  1      1  seq
........................................................guuugugaAauucggccuaaga..............................  8      1  seq
........................................................guuugugUuauucggccuaaga..............................  4      1  seq
........................................................gCuugugauauucggccuaaga..............................  1      1  seq
........................................................guuugugauauucgCccuaaga..............................  1      1  seq
........................................................guuugugauauucUgccuaaga..............................  4      1  seq
........................................................guuugugauauucgUccuaaga..............................  1      1  seq
........................................................gNuugugauauucggccuaaga..............................  2      1  seq
........................................................guuugugauauucggccuGaga..............................  4      1  seq
........................................................guuugugauauucggccuaaUa..............................  1      1  seq
........................................................guuugugauauucgAccuaaga..............................  5      1  seq
........................................................guuugugauaGucggccuaaga..............................  2      1  seq
........................................................guuugugauauucCgccuaaga..............................  1      1  seq
```

```
                    Mature                              Star
auguuuucucgcgugaaaucaacggccgcauauuauaaaucacuuauuugaauguggguuugugauauucggccuaagauuaccacucguaaugcugcuaacaauaucugauu
........................................................guuugGgauauucggccuaaga..................................   2      1     seq
........................................................guuugugauauucggccuaUga..................................   2      1     seq
........................................................guuugugauauucggccuaagaC.................................   1      1     seq
........................................................guuugugauauucggccuaagaA.................................   442    1     seq
........................................................guuugugauauucggccuaagau.................................   26     0     seq
........................................................guuugugauauucggccuaagauu................................   2      0     seq
........................................................guuugugauauucggccuaagaAu................................   100    1     seq
.........................................................uuugAgauauucggccua.....................................   1      1     seq
.........................................................uuugugauauucggccua.....................................   2      0     seq
.........................................................uuugAgauauucggccuaag...................................   1      1     seq
.........................................................uuugugauauucggccuaag...................................   11     0     seq
.........................................................uuugugauauucggccuaaga..................................   1645   0     seq
.........................................................uuugugauauucggccuaaAa..................................   1      1     seq
.........................................................uuugugauauucgUccuaaga..................................   1      1     seq
.........................................................uuugugauauucggccuaUga..................................   1      1     seq
.........................................................uuugugauauucAgccuaaga..................................   1      1     seq
.........................................................uuugugauauucgAccuaaga..................................   1      1     seq
.........................................................uuAgugauauucggccuaaga..................................   1      1     seq
.........................................................Guugugauauucggccuaaga..................................   3      1     seq
.........................................................uuugugauauAcggccuaaga..................................   1      1     seq
.........................................................uuugAgauauucggccuaaga..................................   1      1     seq
.........................................................uuugugauauuAggccuaaga..................................   2      1     seq
.........................................................uuuguAauauucggccuaaga..................................   1      1     seq
.........................................................uuugugauaCucggccuaaga..................................   1      1     seq
.........................................................uuugugauauucggGcuaaga..................................   1      1     seq
.........................................................Auugugauauucggccuaaga..................................   4      1     seq
.........................................................uuugugauauuUggccuaaga..................................   1      1     seq
.........................................................uuugugauauucggUcuaaga..................................   1      1     seq
.........................................................uuugugauauucggccuaagau.................................   1      0     seq
.........................................................uuugugauauucggccuaagaA.................................   24     1     seq
.........................................................uuugugauauucggccuaagaAu................................   38     1     seq
..........................................................uugugauauucggccuaagaAu................................   1      1     seq
```
